# How Quality Control Systems AID Sec-Dependent Protein Translocation

**DOI:** 10.3389/fmolb.2021.669376

**Published:** 2021-04-13

**Authors:** Chen Jiang, Max Wynne, Damon Huber

**Affiliations:** School of Biosciences and the Institute for Microbiology and Infection, University of Birmingham, Birmingham, United Kingdom

**Keywords:** Sec, protein translocation, quality control, protein targeting, molecular chaperones, proteases

## Abstract

The evolutionarily conserved Sec machinery is responsible for transporting proteins across the cytoplasmic membrane. Protein substrates of the Sec machinery must be in an unfolded conformation in order to be translocated across (or inserted into) the cytoplasmic membrane. In bacteria, the requirement for unfolded proteins is strict: substrate proteins that fold (or misfold) prematurely in the cytoplasm prior to translocation become irreversibly trapped in the cytoplasm. Partially folded Sec substrate proteins and stalled ribosomes containing nascent Sec substrates can also inhibit translocation by blocking (i.e., “jamming”) the membrane-embedded Sec machinery. To avoid these issues, bacteria have evolved a complex network of quality control systems to ensure that Sec substrate proteins do not fold in the cytoplasm. This quality control network can be broken into three branches, for which we have defined the acronym “AID”: (i) *avoidance* of cytoplasmic intermediates through cotranslationally channeling newly synthesized Sec substrates to the Sec machinery; (ii) *inhibition* of folding Sec substrate proteins that transiently reside in the cytoplasm by molecular chaperones and the requirement for posttranslational modifications; (iii) *destruction* of products that could potentially inhibit translocation. In addition, several stress response pathways help to restore protein-folding homeostasis when environmental conditions that inhibit translocation overcome the AID quality control systems.

## Graphical Abstract

Overview of the AID quality control pathways.

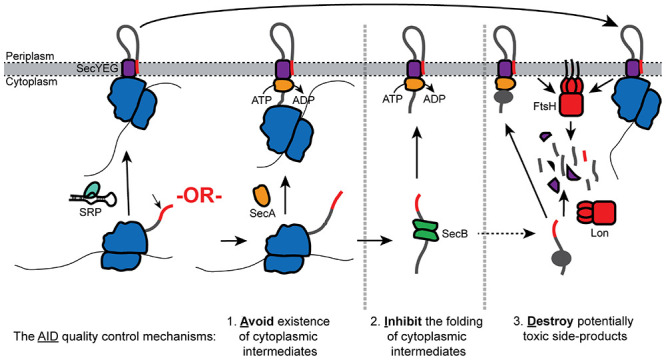

## Introduction

In bacteria, a significant subset of proteins is localized to the cell envelope, which in the Gram-negative bacterium *Escherichia coli* consists of the cytoplasmic membrane, the outer membrane, and the soluble compartment sandwiched in-between known as the periplasm (Tsirigotaki et al., 2017; Cranford-Smith and Huber, 2018). For most of these proteins, the Sec machinery is responsible for the first step in their correct localization, which is translocation across the cytoplasmic membrane (Cranford-Smith and Huber, 2018). Protein substrates of this machinery must be in an unfolded conformation in order to pass through the membrane-embedded Sec machinery and across the cytoplasmic membrane (Randall and Hardy, 1986; Tani et al., 1989; Hardy and Randall, 1991; Uchida et al., 1995). However, many Sec substrate proteins are capable of folding, misfolding, or aggregating in the cytoplasm, and the proteins that do fold (or misfold) prior to translocation become irreversibly trapped in the cytoplasm (Randall and Hardy, 1986;

Kumamoto and Gannon, 1988). Consequently, protein folding presents a predicament for Sec-dependent protein translocation: Sec substrate proteins must fold at their final destination to carry out their function, but premature folding prevents their correct localization.

The two core components of the bacterial Sec machinery are SecYEG and SecA (Cranford-Smith and Huber, 2018). During translocation, substrate proteins pass through a protein-conducting channel in the cytoplasmic membrane formed by the integral cytoplasmic membrane protein (IMP) SecY, which is stabilized by the IMPs SecE and SecG (SecYEG) (Van den Berg et al., 2004; Cannon et al., 2005). The requirement for unfolded proteins is a consequence of the dimensions of the SecYEG channel: proteins must be almost completely unfolded in order to pass through the central constriction in the channel (Randall and Hardy, 1986; Tani et al., 1989; Uchida et al., 1995; Gumbart and Schulten, 2006; Tian and Andricioaei, 2006; Cranford-Smith and Huber, 2018). SecA is an ATPase that drives the translocation of substrate proteins through SecYEG through repeated rounds of ATP binding and hydrolysis (Lill et al., 1989; Brundage et al., 1990). Several mechanisms have been proposed for SecA-mediated translocation and reviewed elsewhere (Cranford-Smith and Huber, 2018; Allen et al., 2020; Catipovic and Rapoport, 2020). In addition to SecYEG and SecA, a number of evolutionarily conserved IMPs, including SecD, SecF, YidC, and YajC, form a supercomplex with SecYEG *in vivo* known as the holotranslocon and assist the core Sec machinery (Schulze et al., 2014; Botte et al., 2016; Komar et al., 2016).

Folding (or misfolding) of a Sec substrate protein in the cytoplasm prior to translocation inhibits Sec-dependent translocation both directly and indirectly. Most obviously, folding inhibits translocation of the protein itself (Randall and Hardy, 1986; Teschke et al., 1991; Huber et al., 2005b). However, folded proteins that are partially translocated across the membrane can become stuck and block (or “jam”) the SecYEG channel (Bieker et al., 1990). The jammed SecYEG is rapidly degraded, which can inhibit translocation indirectly when the jamming occurs on a large scale (van Stelten et al., 2009). Finally, substrate proteins that accumulate in the cytoplasm competitively inhibit translocation by making non-productive interactions with the cytoplasmic Sec machinery (Valent et al., 1997; Drew et al., 2003; Wagner et al., 2007; Klepsch et al., 2011). Inhibition of translocation also results in the accumulation of misfolded Sec substrates in the cytoplasm, which disturbs the protein-folding homeostasis of the cell (Wild et al., 1992, 1993). Cells have evolved a complex network of quality control systems to prevent or address these issues. The mechanisms of this quality control network can be divided into three branches, which we refer to by the acronym “AID.”

1. Mechanisms that **avoid** the existence of unfolded cytoplasmic intermediates through efficient delivery of newly synthesized substrate proteins to the Sec machinery,
2. Mechanisms that **inhibit** the folding of Sec substrate proteins that transiently reside in the cytoplasm,
3. Mechanisms that result in the **destruction** of products that could inhibit translocation.

In this review, we focus on the quality control network of *E. coli* because it is the most extensively investigated bacterial system. However, because the basic mechanism of bacterial protein translocation is evolutionarily conserved, the quality control networks of other bacterial species will fit the AID rubric even when there are some additional or absent mechanisms.

## Avoidance of Cytoplasmic Intermediates Through Cotranslational Targeting

**FIGURE 1:**
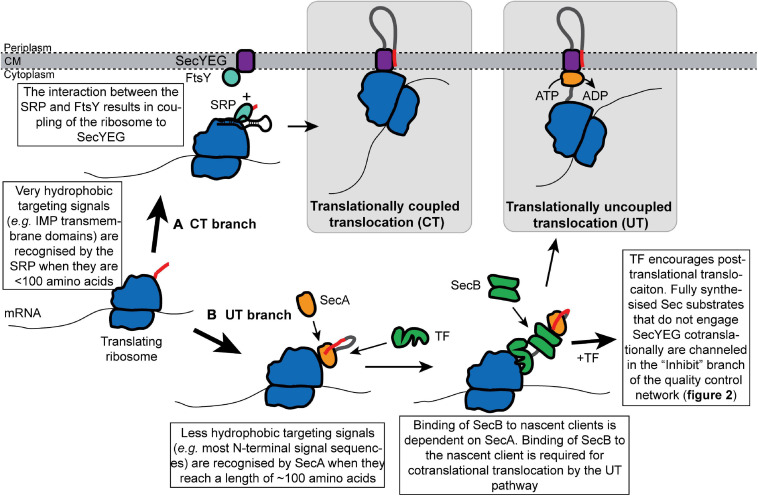
Cotranslational recognition of nascent Sec substrate proteins by the SRP or SecA allows cells to avoid the existence of cytoplasmic intermediates. Nascent Sec substrates are recognized by ribosome-bound SRP or SecA. **(A)** In translationally coupled translocation (CT), the SRP recognizes the targeting signal of a subset of Sec substrates (primarily IMPs) from an early stage in protein synthesis. The SRP targets the translating ribosome to SecYEG by interacting with its receptor protein FtsY at the membrane, which results in the binding of the translating ribosome to SecYEG and direct coupling of translocation to protein synthesis. **(B)** Sec substrates that fail to be recognized by the SRP are targeted for translationally uncoupled translocation (UT) by SecA. SecA recognizes the targeting signal of a nascent Sec substrate when it is about 120 amino acids from the peptidyl transferase site in the ribosome. SecA then recruits SecB to the nascent chain. Upon recruitment of SecB, nascent substrates of the UT pathway can either engage SecYEG cotranslationally or can be held in a translocation-competent conformation by the “Inhibit” branch of the quality control network.

In bacteria, proteins can be transported through SecYEG by one of the two mechanisms: (i) translationally coupled translocation (CT) (Figure 1A) or (ii) translationally uncoupled translocation (UT) (Figure 1B; Rapoport, 2007; Steinberg et al., 2018). During CT, protein translocation is obligately cotranslational: ribosomes are directly bound to SecYEG from an early stage in protein synthesis, which allows the Sec substrates to be synthesized directly into the protein-conducting channel and across the cytoplasmic membrane (Schierle et al., 2003; Jomaa et al., 2016, 2017). Consequently, CT avoids the presence of a cytoplasmic intermediate entirely. During UT, protein translocation can be either co- or post-translational, but it is not directly coupled to protein synthesis (Josefsson and Randall, 1981a, b; Randall, 1983). In addition, many proteins exported by the UT mechanism are fully synthesized before translocation begins (Josefsson and Randall, 1981a, b). In most publications, CT is commonly referred to as the “cotranslational” pathway, while UT is commonly known as the “posttranslational” pathway. However, because UT substrates can engage SecYEG cotranslationally (Josefsson and Randall, 1981a), this terminology is potentially confusing and we have avoided it.

### Cotranslational Targeting to the CT Pathway

Protein substrates of the CT pathway are initially recognized by the signal recognition particle (SRP), a ribonucleoprotein complex that consists of the Ffh protein and the 4.5S SRP-RNA (Figure 1A; Saraogi and Shan, 2014; Steinberg et al., 2018) (An SRP-independent recognition mechanism has also been proposed but is not discussed here; Bibi, 2012). The SRP binds to the 23S ribosomal RNA on the large subunit of the ribosome near the opening of the polypeptide exit channel at a site that also includes the ribosomal proteins uL23, uL24, and uL29 (Gu et al., 2003; Halic et al., 2006; Schaffitzel et al., 2006; Jomaa et al., 2016, 2017). Binding at this site allows Ffh to sample nascent chains and bind to the exposed targeting signal of its substrate proteins just as they emerge from the ribosome (Jomaa et al., 2016; Denks et al., 2017). In eukaryotes, binding of the SRP to a targeting signal induces a transient translational pause, which is relieved upon transfer to the membrane-bound machinery (Walter and Johnson, 1994). The *Bacillus subtilis* SRP may also induce translational pausing (Beckert et al., 2015). However, SRP-induced translational pausing has not been observed in *E. coli*, and the *E. coli* SRP-RNA lacks the domain that induces pausing in other species (Powers and Walter, 1997). Ffh targets the translating ribosome to the cytoplasmic membrane by interacting with its receptor protein FtsY, and coordinated guanosine diphosphate (GTP) hydrolysis by Ffh and FtsY results in coupling of the translating ribosome to SecYEG (Zhang et al., 2010; Saraogi and Shan, 2014).

### Cotranslational Targeting to the UT Pathway

Nascent protein substrates of the UT pathway are recognized cotranslationally by SecA (Figure 1B; Huber et al., 2017; Wang et al., 2017). SecA binds to the ribosome near the opening to the polypeptide exit channel at a site near the SRP binding site, which includes the ribosomal proteins uL23 and uL29 (Huber et al., 2011; Singh et al., 2014; Jamshad et al., 2019; Wang et al., 2019). A portion of SecA may also protrude into the polypeptide exit channel when it is bound to the ribosome (Knupffer et al., 2019). Mutations that disrupt the interaction between SecA and the ribosome cause a defect in UT *in vivo* (Huber et al., 2011).

SecA binds a wide range of nascent Sec substrate proteins *in vivo* (Chun and Randall, 1994; Huber et al., 2017; Wang et al., 2017). SecA can bind to nascent polypeptides when they reach a length of approximately 120 amino acids (Huber et al., 2017), which is consistent with the positioning of SecA in cryo-electron microscopic (EM) structures of the SecA-ribosome complex (Singh et al., 2014; Wang et al., 2019). Binding to nascent polypeptides requires a conformation change in SecA: the C-terminal tail of SecA autoinhibits the protein when it is not bound to a substrate protein (Gelis et al., 2007; Jamshad et al., 2019). Interaction of SecA with the ribosome destabilizes this autoinhibited conformation and activates SecA to binding to nascent substrates (Jamshad et al., 2019). SecA then recruits the molecular chaperone SecB to the nascent polypeptide chain (see the section on SecB below for more details) (Huber et al., 2017). Recruitment of SecB is required for the cotranslational targeting to SecYEG (Kumamoto and Gannon, 1988; Huber et al., 2017). Some early studies suggested that SecB can directly recognize nascent polypeptides (Kumamoto and Gannon, 1988; Kumamoto and Francetic, 1993; Fekkes et al., 1998), and binding to SecB can activate SecA to bind to substrate proteins (Gelis et al., 2007). However, binding of SecB to nascent clients is dependent on SecA *in vivo*, suggesting that it is SecA that normally recognizes nascent substrates of the UT pathway (Huber et al., 2017).

### Sorting to the CT and UT Pathways

Sec substrate proteins are recognized by virtue of an internally encoded targeting signal (Bassford and Beckwith, 1979; Ulbrandt et al., 1997; Schierle et al., 2003; Hegde and Bernstein, 2006). In the case of IMPs, this targeting signal is a transmembrane helix (or, occasionally, multiple transmembrane helices) (Ulbrandt et al., 1997; Schibich et al., 2016). For outer membrane proteins (OMPs), soluble periplasmic proteins (PPs), and lipoproteins (LPs), the targeting signal is an N-terminal signal sequence, which is proteolytically removed from the protein during translocation (von Heijne, 1990; Hegde and Bernstein, 2006). Most IMPs are targeted to the CT pathway (Ulbrandt et al., 1997; Schibich et al., 2016), and although the CT pathway does recognize a small subset of cleavable signal sequences, most OMPs, PPs, and LPs are targeted to the UT pathway (Huber et al., 2005a). The distinguishing feature of the targeting signals recognized by the CT pathway is that they are more hydrophobic than those that target proteins to the UT pathway (Lee and Bernstein, 2001; Schierle et al., 2003; Huber et al., 2005a; Schibich et al., 2016; Cranford-Smith and Huber, 2018). Mutations that increase the hydrophobicity of a UT signal sequence can re-route translocation to the CT pathway (Lee and Bernstein, 2001; Bowers et al., 2003).

Sorting to the CT or UT pathway appears to be determined by a triaging mechanism: if a targeting signal is sufficiently hydrophobic, the substrate protein will be channeled into the CT pathway, while proteins containing less hydrophobic targeting signals are channeled into the UT pathway by default (Lee and Bernstein, 2001; Schierle et al., 2003). The physiological basis for the evolution of a bifurcated targeting pathway is likely complex. For example, some proteins may be targeted to the CT pathway because they are prone to aggregation in the cytoplasm (such as IMPs) (Ulbrandt et al., 1997). Others may fold too rapidly to be exported by the UT pathway (Huber et al., 2005a) or are toxic in the cytoplasm. The choice of pathway can also affect the folding pathway of a protein in the periplasm (Kadokura and Beckwith, 2009). However, high levels of CT could potentially be toxic under conditions that inhibit translocation elongation (van Stelten et al., 2009), and the rate of CT is probably inherently slower than that of UT because it is limited by the rate of translocation elongation (Pugsley, 1993; Cranford-Smith and Huber, 2018). Finally, the existence of two pathways may allow the UT pathway to serve as a backup pathway for CT when the CT pathway is defective (Lee and Bernstein, 2001; Schierle et al., 2003; Zhao et al., 2021).

### Trigger Factor Delays Delivery of UT Substrate Proteins to SecYEG

The ribosome-associated molecular chaperone Trigger Factor (TF) delays the delivery of many nascent Sec substrates to SecYEG (Lee and Bernstein, 2002; Ullers et al., 2007; Oh et al., 2011). TF binds to the ribosome near the polypeptide exit channel at a site that includes uL23 and hunches over the opening to the channel (Kramer et al., 2002; Ferbitz et al., 2004). This ribosome-binding activity facilitates the interaction of TF with nascent polypeptides (Kramer et al., 2002, 2004, 2019). Although SecA and TF bind to similar sites on the ribosome (Kramer et al., 2002; Huber et al., 2011), binding is not mutually exclusive and both proteins can bind to the same nascent chain simultaneously (Huber et al., 2017). TF binds to hydrophobic patches in non-native nascent polypeptides with relatively low specificity and can begin to interact with nascent polypeptides when they reach a length of approximately 110 amino acids *in vivo* (Patzelt et al., 2001; Kramer et al., 2002; Oh et al., 2011; Bornemann et al., 2014). The binding of TF to nascent chains is thought to delay the folding of most nascent polypeptides, which facilitates the correct folding of cytoplasmic proteins by preventing off-pathway folding intermediates (Deuerling et al., 1999; Kramer et al., 2004, 2019; Hoffmann et al., 2006; Kaiser et al., 2006; Merz et al., 2008; Martinez-Hackert and Hendrickson, 2009; Nilsson et al., 2016; Liu et al., 2019).

TF was initially identified in biochemical screens for proteins that promote Sec-dependent protein translocation (Crooke and Wickner, 1987; Crooke et al., 1988; Lill et al., 1988; Lecker et al., 1989), and ribosome profiling experiments indicate that TF binds to many nascent Sec substrates, particularly OMPs (Oh et al., 2011). Strains deficient in TF have a mild outer membrane biogenesis defect (Oh et al., 2011), and TF can enhance translocation *in vitro* (Crooke et al., 1988; De Geyter et al., 2020), which has led to the suggestion that TF can *inhibit* the folding of Sec substrates (see below). However, strains lacking TF do not have an obvious defect in Sec-dependent protein translocation (Lee and Bernstein, 2002). Indeed, mutations that disrupt the gene encoding TF (*tig*) suppress many translocation defects by allowing nascent UT substrates to engage SecYEG cotranslationally (Lee and Bernstein, 2002; Ullers et al., 2007; Oh et al., 2011), suggesting that TF prevents nascent Sec substrates from engaging SecYEG cotranslationally. It has been suggested that TF could compete with the SRP for binding to substrate proteins (Eisner et al., 2003, 2006; Ariosa et al., 2015). However, a growing body of evidence suggests that TF does not play a role in the choice of pathway (i.e., CT vs. UT); rather, the binding of TF to nascent UT substrates prevents them from engaging SecYEG cotranslationally (Lee and Bernstein, 2002; Ullers et al., 2007; Huber et al., 2017). Thus, the role of TF in Sec-dependent translocation is to enhance the bifurcation of the two translocation pathways, potentially for the reasons discussed above.

## Inhibition of Protein Folding of Sec Substrates in the Cytoplasm

**FIGURE 2:**
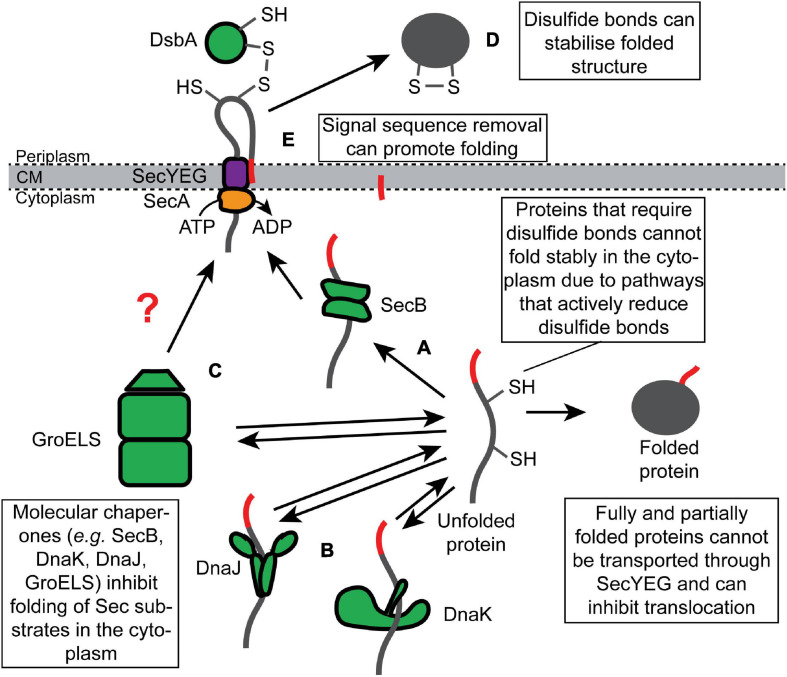
Inhibition of the folding of synthesized Sec substrate proteins by molecular chaperones and posttranslational modifications. Fully synthesized Sec substrate proteins (or domains of Sec substrate proteins) are prevented from folding stably in the cytoplasm by molecular chaperones, such as **(A)** SecB, **(B)** DnaK or DnaJ, and **(C)** GroELS. Alternatively, many Sec substrate proteins require posttranslational modification that can only be made in the periplasm to fold stably, such as **(D)** disulfide bonds and **(E)** proteolytic removal of the N-terminal signal sequence.

Because many substrates of the UT pathway are fully synthesized (or nearly fully synthesized) before they engage SecYEG, these proteins have the potential to fold (or misfold) in the cytoplasm prior to translocation. Cells prevent the premature folding of Sec substrate proteins *via* two mechanisms: (i) molecular chaperones, which bind to unfolded Sec substrate proteins and hold them in a translocation-competent conformation in the cytoplasm (Figures 2A–C); and (ii) requirements of posttranslational modifications that can only be made upon translocation for stable folding (Figures 2D,E). By convention, we refer to proteins as “clients” of molecular chaperones and “substrates” of the Sec machinery.

### Inhibition of Folding by SecB

SecB is a tetrameric molecular chaperone that binds to a subset of unfolded substrates of the UT pathway and prevents them from folding in the cytoplasm (Figure 2A; Collier et al., 1988; Hartl et al., 1990; Zhou and Xu, 2003). Mutations disrupting the *secB* gene cause defective translocation of this subset *in vivo* (Kumamoto and Beckwith, 1983, 1985; Baars et al., 2006). SecB binds to hydrophobic patches in its non-native client proteins in an ATP-independent fashion (Randall and Hardy, 2002; Huang et al., 2016). SecB binds to clients with relatively low specificity *in vitro* (Randall et al., 1998a, b; Knoblauch et al., 1999) but with high selectivity *in vivo* (Kumamoto and Beckwith, 1985; Kumamoto and Francetic, 1993). This difference could be explained by the dependence of SecB on SecA for binding to nascent substrates *in vivo* since SecA does display an increased affinity for proteins containing N-terminal signal sequences (Kebir and Kendall, 2002; Gouridis et al., 2009; Huber et al., 2017). SecB can bind to full-length proteins and target them for translocation in a reconstituted system *in vitro* (Fekkes et al., 1998). However, it is not clear whether this is also the case *in vivo*. If so, recognition likely requires clients to fold slowly enough for SecB to bind cooperatively to multiple low-affinity binding sites (Hardy and Randall, 1991; Randall et al., 1998b).

SecB ultimately delivers its client proteins to the translocation machinery by binding to SecA (Gannon and Kumamoto, 1993; Fekkes et al., 1998). The interaction between SecA and SecB is driven by at least two sites of interaction (Woodbury et al., 2000; Randall et al., 2004; Crane et al., 2005; Randall and Henzl, 2010): first, the small metal-binding domain (MBD) at the extreme C-terminus of SecA binds to an evolutionarily conserved binding surface on SecB (Fekkes et al., 1997, 1999; Zhou and Xu, 2003); second, the C-terminal α-helix of SecB interacts with the catalytic core of SecA (Woodbury et al., 2000; Randall et al., 2004; Randall and Henzl, 2010). SecB transfers its client proteins to SecA by destabilizing the autoinhibited conformation of SecA (Gelis et al., 2007). Because the steady state affinity of SecA for non-native translocation-competent Sec substrates is at least an order of magnitude lower than that of SecB, the transfer of client proteins from SecB to SecA also likely requires a conformation change in SecB that reduces the affinity of SecB for its client (Randall et al., 1998b; Woodbury et al., 2000; Gouridis et al., 2009). Nearly all α-,β-, and γ-*Proteobacteria* species contain a SecB homolog, but SecB is conspicuously absent from many bacterial phylogenetic groups (even those containing a SecA protein with an MBD) (van der Sluis and Driessen, 2006; Jamshad et al., 2019). However, some phylogenies contain proteins that are structurally related to SecB and that could have a similar function, suggesting that the presence of SecB-like proteins could be a universal feature of Sec-dependent protein translocation in bacteria (Sala et al., 2014).

Although SecB is not essential for viability in *E. coli* (Kumamoto and Gannon, 1988; Shimizu et al., 1997), deficiencies in SecB-dependent quality control cause collateral defects in protein translocation and protein-folding homeostasis. For example, mutations that inactivate the *secB* gene cause defects in the translocation of proteins that do not normally bind to SecB *in vivo* (Francetic and Kumamoto, 1996), suggesting that a lack of quality control causes a translocation defect that has knock-on consequences for non-client proteins. Mutations in *secB* also result in induction of the heat shock response (Wild et al., 1993), indicating a perturbation in the protein-folding homeostasis. Deletion of the *secB* gene causes a cold-sensitive growth defect (Shimizu et al., 1997), which is likely caused by the combined effect on protein translocation and the protein-folding homeostasis (Altman et al., 1991; Ullers et al., 2007; Sakr et al., 2010).

### Inhibition of Folding by General Chaperone Systems

Two general chaperone systems, the DnaK/DnaJ (Figure 2B) and the GroEL/GroES (Figure 2C) systems, have been implicated in Sec-dependent protein translocation. Unlike SecB, whose role is normally restricted to Sec-dependent translocation (Kumamoto and Beckwith, 1985; Kumamoto and Francetic, 1993), the DnaK/DnaJ and GroEL/GroES systems assist the folding of a wide range of soluble cytoplasmic proteins (Kim et al., 2013; Dahiya and Buchner, 2019). In the DnaK/DnaJ system, DnaJ (Hsp40) binds to non-native or misfolded client proteins and delivers them to the ATPase DnaK (Hsp70), and this interaction stimulates a conformational change in DnaK, driven by ATP hydrolysis, that promotes folding of the client protein (Rosenzweig et al., 2019). GrpE-stimulated nucleotide exchange releases the client protein and promotes refolding (Rosenzweig et al., 2019). Mutations disrupting the DnaK chaperone system cause a defect in the translocation of a subset of Sec substrate proteins and cause growth defects when combined with mutations that inactivate *secB* (Altman et al., 1991; Wild et al., 1992, 1996; Lee and Bernstein, 2002; Ullers et al., 2007), suggesting that DnaK can compensate for the loss of SecB. Overexpression of DnaK or DnaJ individually can suppress these defects and even enhance the efficiency with which some proteins are exported (Phillips and Silhavy, 1990; Sakr et al., 2010). However, overexpression of both proteins simultaneously cannot suppress the phenotype of a *secB* mutant (Sakr et al., 2010), suggesting that DnaK and DnaJ promote protein translocation by holding Sec substrates in a translocation-competent conformation (Figure 2B).

Several early studies suggested that the GroEL/GroES chaperone system could also assist the Sec machinery (Crooke et al., 1988; Kusukawa et al., 1989; Lecker et al., 1989). In this system, GroEL binds to misfolded client proteins, and the binding of GroES to GroEL stimulates an ATP-dependent conformational change in GroEL that promotes protein folding (Horwich et al., 2006). GroEL binds to non-native Sec substrates *in vitro* (Lecker et al., 1989), and mutants that are deficient in GroEL or GroES are defective in the translocation of UT substrates (Kusukawa et al., 1989), suggesting that GroEL/GroES can assist Sec-dependent translocation. In support of this notion, the overproduction of GroEL enhances the translocation efficiency of LamB-LacZ (Phillips and Silhavy, 1990). In addition, GroEL localizes to the cytoplasmic membrane, and localization is dependent on SecA (Bochkareva et al., 1998), suggesting that GroEL could bind to non-native translocation-competent Sec substrates and target them to SecA (Figure 2C). However, the involvement of GroEL/GroES in protein translocation is debated (Altman et al., 1991).

### Posttranslational Modifications That Facilitate Protein Folding

The Sec quality control network has also exploited some posttranslational modifications that facilitate protein folding or stabilize the final folded structure, which can only be made upon protein translocation. For example, disulfide bonds create covalent links between cysteine amino acid side chains that stabilize the tertiary structure of the protein (Manta et al., 2019). In *E. coli*, disulfide bonds are formed by the periplasmic Dsb machinery, which passes the electrons from the oxidized cysteines in the client protein to a reduced quinone in the cytoplasmic membrane *via* a series of disulfide exchange reactions (Landeta et al., 2018; Manta et al., 2019). Many proteins, such as alkaline phosphatase (PhoA), require structural stabilization from disulfide bonds in order to fold into an active conformation (Figure 2D; Sone et al., 1997). A highly redundant network of thiol redox pathways actively reduces disulfide bonds in the cytoplasm (Ezraty et al., 2017), which prevents proteins like PhoA from folding stably while they transiently reside in the cytoplasm. In some bacteria, the folding of exported proteins can also be stabilized by other types of covalent linkages between amino acid side chains, such as isopeptide bonds (Kang et al., 2007; Kang and Baker, 2011).

A second posttranslational modification that can facilitate folding is proteolytic removal of the N-terminal signal sequence. The signal sequences of some proteins, such as maltose-binding protein (MBP) and ribose binding protein (RBP), slow the folding of their cognate proteins (Park et al., 1988), and the reduction in the rate of folding is required for efficient interaction with SecB (Liu et al., 1989). However, signal sequences are removed during translocation by signal peptidase (Josefsson and Randall, 1981a, b; von Heijne, 1990; Hegde and Bernstein, 2006; Figure 2E). Biophysical experiments suggest that the signal sequence of MBP slows MBP folding by binding to the hydrophobic core of the non-native protein (Beena et al., 2004), and the conserved architecture of signal sequences suggest that this anti-folding activity may be a general property (von Heijne, 1990). If so, the effect of the signal sequence on folding is moderate since the MBP signal sequence cannot sufficiently retard the folding of at least two normally cytoplasmic proteins (thioredoxin-1 and DARPin) to allow efficient translocation by the UT pathway (Schierle et al., 2003; Steiner et al., 2006).

## Destruction of Products That Inhibit Protein Translocation

Proteins that escape the “Avoid” and “Inhibit” branches of the Sec quality control network are “Destroyed” by proteases. Two cytoplasmic proteases, Lon and FtsH, appear to be responsible for most of the turnover of potentially toxic Sec substrates in the cytoplasm (van Stelten et al., 2009; Sakr et al., 2010). Both Lon and FtsH are general proteases that belong to the AAA^+^ (ATPase associated with cellular activities) family of proteases, which also includes ClpXP, ClpAP, and HslUV proteases (Sauer and Baker, 2011). AAA^+^ proteases contain ATPase motor domains that unfold substrate proteins and feed them into the proteolytic active site of a protease module (Sauer and Baker, 2011). In addition, a cytoplasmic peptidase, PrlC, with specificity for N-terminal signal sequences assists Sec-dependent protein translocation *in vivo* (Conlin et al., 1992).

### Destruction of Cytoplasmic Sec Substrates by Lon Protease

**FIGURE 3:**
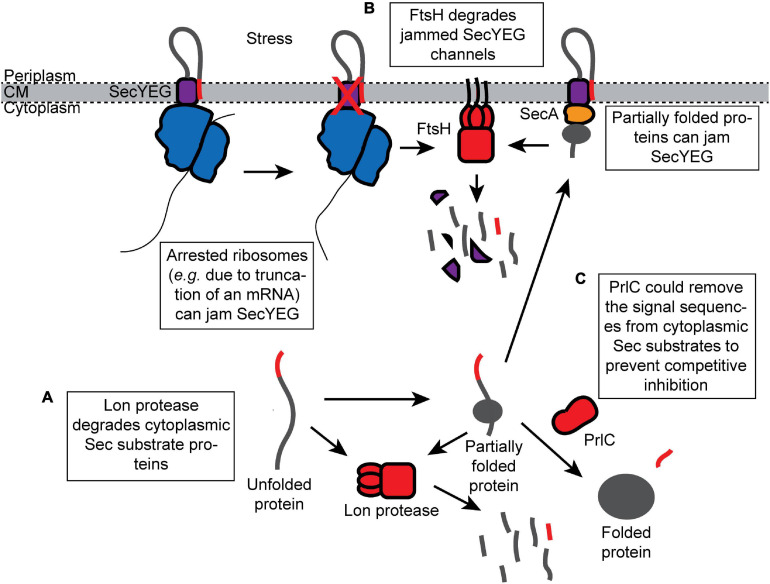
Proteolytic destruction of products that are detrimental to protein translocation. **(A)** Lon protease prevents mislocalized Sec substrates from inhibiting the Sec machinery by turning over Sec substrates that accumulate in the cytoplasm. **(B)** FtsH degrades SecYEG channels that have been jammed (e.g., by arrested ribosomes synthesizing Sec substrate proteins). **(C)** The peptidase PrlC could potentially remove the signal sequences from the Sec substrate proteins that have accumulated in the cytoplasm and prevent them from competitively inhibiting translocation.

Lon protease degrades missorted Sec substrate proteins that accumulate in the cytoplasm (Figure 3A). For example, Lon degrades mutant M13 procoat protein when it is mislocalized to the cytoplasm (Kuhn et al., 1986). In addition, mutations in the *prlF* gene can enhance the translocation of Sec substrate proteins *in vivo* by influencing the activity of Lon (Kiino et al., 1990; Snyder and Silhavy, 1992; Minas and Bailey, 1995). PrlF is the antitoxin component of a toxin–antitoxin system in *E. coli* and is normally degraded by Lon protease (Schmidt et al., 2007). Mutations that inactivate Lon suppress the cold-sensitive viability defect caused by a Δ*secB* deletion mutation but also cause the accumulation of aggregated Sec substrates in the cytoplasm (Sakr et al., 2010), suggesting that Lon normally degrades Sec substrate proteins that escape the other quality control pathways.

### Destruction of Jammed SecYEG Complexes by FtsH

FtsH is a membrane-anchored protease that turns over uncomplexed, misfolded, or jammed SecY channels (Figure 3B; Kihara et al., 1995, 1996; van Stelten et al., 2009). FtsH-mediated degradation of SecY can be inhibited by the expression of YccA (van Stelten et al., 2009). It has been suggested that FtsH-mediated degradation clears SecY channels blocked by the arrested ribosomes translating Sec substrate proteins (e.g., due to truncated mRNAs), which may be required to recycle the arrested ribosome (van Stelten et al., 2009). The prevalence and redundancy of ribosome rescue systems suggest that translational arrest is relatively common (Keiler, 2015). In addition, cells deficient in FtsH are defective for Sec-dependent protein translocation (Akiyama et al., 1994), suggesting that rapid clearance of “dead” SecYEG complexes is required to maintain the efficiency of translocation under normal growth conditions.

### Other Peptidases

A cytoplasmic peptidase, PrlC (oligopeptidase A), also assists Sec-dependent protein translocation *in vivo* (Conlin et al., 1992; Kato et al., 1992). Certain mutations in *prlC* enhance the translocation of Sec substrate proteins containing defective signal sequences *in vivo* (Emr and Bassford, 1982; Trun and Silhavy, 1987). Biochemical studies suggest that PrlC has specificity for Sec signal sequences (Novak and Dev, 1988; Conlin et al., 1992). However, the molecular mechanism is not known. One possibility is that PrlC degrades free, proteolytically processed signal sequences, which competitively inhibit protein translocation. Alternatively, PrlC could remove signal sequences from Sec substrates that are mislocalized to the cytoplasm, which is an idea that is supported by the accumulation of N-terminally processed Sec substrate in the cytoplasm of some *prlC* mutants (Figure 3C; Trun and Silhavy, 1989).

## Cell Stress Responses That Restore Protein-Folding Homeostasis

Environmental stresses that inhibit translocation can cause a detrimental feedback loop that can overcome the AID quality control systems and disturb protein-folding homeostasis. In an example scenario, Sec substrate proteins that accumulate in the cytoplasm could partially fold and cause wide-scale jamming of SecYEG, which would result in the quantitative destruction of SecY by FtsH, enhancing the accumulation of Sec substrate proteins in the cytoplasm (Oliver et al., 1990; Wagner et al., 2007; van Stelten et al., 2009; Klepsch et al., 2011). In *E. coli*, there are at least two stress response pathways that can break this cycle: the σ^32^ pathway and the Cpx pathway (Wild et al., 1993; Cosma et al., 1995). σ^32^ is an alternative sigma factor that recognizes the transcriptional promoters of genes involved in adapting to conditions that perturb protein-folding homeostasis, and the σ^32^ pathway is induced by the accumulation of unfolded and misfolded proteins in the cytoplasm (Roncarati and Scarlato, 2017). Defects in Sec-dependent protein translocation (e.g., caused by mutations in *secB*) result in the accumulation of unfolded or misfolded Sec substrate proteins in the cytoplasm and induction of the σ^32^ pathway (Wild et al., 1992, 1993). σ^32^ controls expression of many proteins that are involved in the AID quality control network (e.g., DnaK/DnaJ, GroELS, PrlC, Lon, and FtsH among others), and its induction can suppress defects caused by inhibition of Sec-dependent protein translocation (Grossman et al., 1987; Altman et al., 1991). In addition, the regulatory circuit that governs the induction of the σ^32^ pathway incorporates signals from FtsH (Tomoyasu et al., 1995) and the SRP (Lim et al., 2013), suggesting that translocation defects are a physiological source of disruptions in protein-folding homeostasis.

Induction of the Cpx pathway suppresses the toxicity caused by jamming of SecYEG (Cosma et al., 1995; Pogliano et al., 1997). The Cpx pathway is induced by conditions that disturb protein-folding homeostasis in the periplasm (Cosma et al., 1995). The suppression of jamming toxicity is due, at least in part, to the inhibition of FtsH by induction of the *yccA* gene (van Stelten et al., 2009).

## Outlook

The number of quality control mechanisms that assist Sec-dependent protein translocation suggests that there is strong evolutionarily pressure to prevent the folding (or misfolding) of Sec substrate proteins in the cytoplasm. However, there are significant gaps in the understanding of this quality control network. For example, the mechanism of CT is not fully understood. SecA is required for efficient CT (Schierle et al., 2003), but it is not clear whether it is involved in the recognition of substrate proteins or the mechanism of translocation across the membrane. In addition, recent work indicating that the SRP is not strictly essential raises fundamental questions about the mechanism of targeting to the CT pathway (Zhao et al., 2021).

It seems likely that there are additional quality control pathways that have not yet been identified. For example, recent work suggests that TF cooperates with the ClpXP protease, raising the possibility that TF could channel misfolded OMPs to ClpXP for destruction (Rizzolo et al., 2021). In addition, there could be previously unidentified components that facilitate these pathways. For example, there are two *E. coli* proteins of unknown function, YecA and YchJ, that contain MBDs that are nearly identical to that of SecA (Cranford-Smith et al., 2020a), and un-peer-reviewed work by Cranford-Smith et al. (2020b) suggests that one of these proteins, YecA, is a molecular chaperone that can interact with SecB. The Pfam database contains at least a dozen other proteins of unknown function that contain SecA-like MBDs in other bacterial species (Finn et al., 2014), raising the possibility that there are many additional accessory Sec components. If so, many of these components could assist with one of the AID mechanisms.

Furthermore, it is possible that there are additional quality control mechanisms that do not fit neatly within the AID rubric. For example, DnaK/DnaJ can work in concert with the AAA^+^ protein ClpB to resolubilize aggregated proteins in the cytoplasm (Schlieker et al., 2004; Rosenzweig et al., 2013; Mogk et al., 2018), raising the possibility that DnaK or another chaperone could cooperate with ClpB to resuscitate folded or aggregated Sec substrates for protein translocation.

Finally, there are still a number of questions about how quality control components distinguish between the substrate and non-substrate proteins. Genetic studies suggest that SecB could directly recognize full-length substrate proteins *in vivo* (Liu et al., 1988), but if so, by what mechanism? Are Sec substrates targeted to Lon protease, or does Lon degrade misfolded or aggregated Sec substrates as part of its normal house-keeping activity (Sauer and Baker, 2011)? How does FtsH distinguish between jammed SecYEG complexes and those that are actively translocating substrate proteins (van Stelten et al., 2009)? Clearly, additional research is required to fully elucidate the quality control network of the Sec machinery.

## Author Contributions

MW wrote drafts of the subsection of molecular chaperones. CJ wrote drafts of the sections on proteases and stress responses. DH conceived the manuscript, wrote drafts of the abstract, introduction and subsection on protein targeting, and was responsible for assembly of the final manuscript. All authors contributed to the writing of this manuscript, contributed to fundamental background research, filling in references and editing the manuscript.

## Conflict of Interest

The authors declare that the research was conducted in the absence of any commercial or financial relationships that could be construed as a potential conflict of interest.
